# Hypoxia-inducible factor 1-alpha: a dual regulator of ferroptosis in ischemic stroke and a promising therapeutic target

**DOI:** 10.3389/fneur.2026.1859829

**Published:** 2026-06-30

**Authors:** Qian Cui, Can Guo, Yinxiang Luo, Dingding Liu, Dong Wang, Zhe Wang, Zhiguang Xu

**Affiliations:** 1 Department of Traditional Chinese Medicine, Cangzhou Medical College, Cangzhou, China; 2 Department of Oncology, First Teaching Hospital of Tianjin University of Traditional Chinese Medicine, Tianjin, China; 3 North China University of Science and Technology, Tangshan, China

**Keywords:** dual roles, ferroptosis, hypoxia-inducible factor 1-alpha, ischemic stroke, therapeutic approaches

## Abstract

Ischemic stroke (IS) is an acute cerebrovascular disorder associated with high morbidity and disability rates. Its pathogenesis is complex and involves multiple forms of cell death. Ferroptosis, a novel form of non-apoptotic cell death, plays a significant role in IS progression. Hypoxia-inducible factor 1-alpha (HIF-1α) is a critical transcription factor in hypoxic responses and exhibits either neuroprotective or neurotoxic effects in IS. Recent studies have identified HIF-1α as a key regulator of ferroptosis after IS. However, whether HIF-1α exerts a dual effect in regulating ferroptosis after IS remains unclear. To address this, we reviewed studies on the regulatory role of HIF-1α in ferroptosis. The findings indicate that HIF-1α regulates ferroptosis through mechanisms involving iron metabolism, lipid peroxidation (LPO), and oxidative stress, thereby exerting both inhibitory and promotive effects. This paradoxical characteristic may be related to dynamic changes in HIF-1α expression across different time points and varying degrees of ischemic severity. We also discuss therapeutic approaches aimed at modulating HIF-1α levels to suppress ferroptosis, including prolyl hydroxylase (PHD) inhibitors, deferoxamine (DFO), dimethyloxallyl glycine (DMOG), 3-(5′-hydroxymethyl-2′-furyl)-1-benzyl indazole (YC-1), propofol, and other natural compounds. Future studies should investigate the dynamic changes of HIF-1α in regulating ferroptosis under different ischemic durations and severities of ischemia to further clarify its dual role and facilitate the development of therapeutic strategies. Nevertheless, clinical translation of these strategies is currently limited by a narrow therapeutic window, potential side effects, and insufficient clinical validation.

## 1 Introduction

Stroke is an acute cerebrovascular disease that includes ischemic stroke (IS) and hemorrhagic stroke. In 2021, there were 11.9 million new stroke cases worldwide (1). Among these, IS accounted for approximately 60–70% of all stroke events globally (2). This imposes a substantial medical burden and economic losses on affected families, and projections indicate that both the number of IS cases and its incidence will continue to increase between 2020 and 2030 (3). IS has therefore become a major cause of disability and mortality worldwide. IS is caused by local obstruction of cerebral blood flow. The resulting ischemic and hypoxic conditions lead to localized brain tissue necrosis and subsequent neurological deficits (4). The most effective treatment for IS is the early and rapid restoration of cerebral blood perfusion. Thrombolytic therapy is a key strategy for achieving early reperfusion. Alteplase is currently the only thrombolytic agent approved by the U. S. Food and Drug Administration (FDA) for the treatment of IS. However, clinical observations have shown that some patients experience progressive worsening rather than improvement of neurological deficits following thrombolytic therapy. This paradoxical phenomenon is referred to as cerebral ischemia–reperfusion injury (CIRI) (5, 6). At present, effective clinical treatments for CIRI remain limited (7), and its underlying mechanisms have not been fully elucidated. Therefore, further investigation of the pathogenesis of IS and the development of novel therapeutic approaches to improve neurological recovery are urgently needed.

It is well established that the pathogenesis of IS involves multiple programmed cell death pathways, including apoptosis, necroptosis, autophagic cell death, and pyroptosis. Increasing evidence suggests that ferroptosis is a newly identified form of non-apoptotic cell death that also plays a critical role in the progression of IS (8). Ferroptosis differs markedly from other forms of cell death, such as autophagy, pyroptosis, necrosis, and apoptosis (9, 10). These differences are primarily reflected in its structural and functional characteristics. Morphologically, ferroptosis is characterized by abnormalities in mitochondrial structure, including increased membrane density, disrupted cristae, and membrane rupture and thickening (11, 12). Functionally, it induces neuronal injury through dysregulation of iron metabolism, lipid peroxidation (LPO), and oxidative stress (13–17). After IS, brain iron levels can rise rapidly, leading to the generation of reactive oxygen species (ROS) and the accumulation of lipid peroxides, thereby triggering ferroptosis (18–20).

Hypoxia-inducible factor 1-alpha (HIF-1α) is a transcription factor activated under hypoxic conditions that regulates the expression of genes involved in oxidative stress, inflammation, and cell death (21). Previous studies have shown that HIF-1α plays a dual role in cerebral ischemia (22–25). After IS, HIF-1α exerts neuroprotective effects by upregulating vascular endothelial growth factor (VEGF), thereby promoting angiogenesis and neurogenesis. HIF-1α also upregulates erythropoietin (EPO) expression, which enhances oxygen transport and cerebral perfusion (26–28). However, some studies have suggested that HIF-1α may exacerbate neurological injury by increasing blood–brain barrier (BBB) permeability and mediating the expression of pro-inflammatory factors during the early stage of IS (29–31). For example, HIF-1α can aggravate post-stroke neural injury through activation of the NOD-like receptor protein 3 (NLRP3) inflammasome, thereby inducing pyroptosis and apoptosis (32). HIF-1α can also enhance inflammatory and oxidative stress responses through the Nuclear Factor kappa-light-chain-enhancer of activated B cells (NF-κB) pathway (33).

Recent studies have indicated that the ferroptosis pathway is regulated by HIF-1α (34, 35). HIF-1α plays a dual role following IS by promoting angiogenesis while also stimulating inflammation, and this duality is likewise reflected in its regulation of ferroptosis. One study showed that, in a rat model of transient middle cerebral artery occlusion (tMCAO), HIF-1α inhibited ferroptosis by downregulating the transcription of long-chain acyl-CoA synthetase family member 4 (ACSL4), thereby exerting a protective effect on brain tissue (36). In contrast, elevated HIF-1α expression can upregulate transferrin receptor 1 (Tfr1) expression in the brain tissue of rats subjected to tMCAO, resulting in pathological iron overload (37).

However, our review of the available studies revealed that no published review has addressed the bidirectional regulatory mechanisms of HIF-1α in ferroptosis during IS or the potential reasons underlying this dual effect. Therefore, this article is the first to summarize the available evidence and proposes that the bidirectional regulatory effect of HIF-1α on ferroptosis may be closely associated with dynamic changes in HIF-1α expression resulting from differences in ischemic duration and IS severity. Notably, there is currently no direct evidence supporting this hypothesis. Future studies should conduct dynamic monitoring across different time points and levels of disease severity to verify the relationship between HIF-1α expression dynamics and ferroptosis regulation.

## 2 Ferroptosis

### 2.1 The naming of ferroptosis

In 1980, Shiro Banna and colleagues found that amino acids entering cells were oxidized in the form of cysteine. They also found that cysteine and glutamate were transported into human epidermal fibroblasts via a specific transporter, where they performed physiological functions (38). In 2003, a novel compound named “erastin,” which was found to induce a type of non-apoptotic and gene-selective cell death, was first identified by Dolma and colleagues (39). Subsequently, Yang et al. discovered that two novel compounds, RSL3 and RSL5, could trigger a non-apoptotic, iron-dependent oxidative cell death. They also showed that cells transformed by oncogenic RAS had elevated iron levels due to increased Tfr1 expression and reduced ferritin heavy chain 1 (FTH1) and ferritin light chain (FTL) expression (40). It was not until 2012 that Dixon et al. coined the term “ferroptosis” and elucidated its morphological, biochemical, and genetic characteristics (41).

### 2.2 The mechanism of IS and ferroptosis

Recent studies have revealed that ferroptosis is a key contributor to the development of IS. The mechanism primarily involves neuronal damage mediated by dysregulated iron metabolism, LPO, and oxidative stress.

#### 2.2.1 Ischemic stroke and disorders of iron metabolism

Iron primarily exists in the human body in the forms of ferrous (Fe^2+^) and ferric (Fe^3+^) ions. Physiologically, iron metabolism is maintained in a state of homeostasis, and the brain remains largely isolated from systemic iron fluctuations. The regulation of iron homeostasis is essential for maintaining normal brain function. This process involves the expression of iron-import proteins, iron-export proteins, and intracellular iron-storage proteins (42). Transferrin (Tf) is a key glycoprotein in plasma that binds and transports Fe^3+^ in the bloodstream. Following binding, the two form a Tf-Fe complex. Tfr1 is a transmembrane glycoprotein that serves as a key mediator of cellular iron uptake and is primarily responsible for transporting the Tf-Fe complex from the extracellular space into the cell via endocytosis (43). After entering the cell via Tfr1, Fe^3+^ is reduced to Fe^2+^ by six-transmembrane epithelial antigen of prostate 3 (STEAP3). As the primary iron-storage protein, ferritin stores excess Fe^2+^ and thereby maintains intracellular iron homeostasis. When iron is needed by the cell, ferritin is degraded via ferritinophagy, which releases Fe^2+^ into the labile iron pool (LIP) for utilization (44). When Fe^2+^ is exported from the cell, a ferrous oxidase such as ceruloplasmin (CP), which is associated with ferroportin 1 (FPN1), can catalyze its oxidation to Fe^3+^ (45). Notably, CP can rapidly oxidize highly toxic Fe^2+^ to less toxic Fe^3+^, which is essential for cerebral iron release, uptake, and homeostasis (46). Subsequently, Fe^3+^ rapidly binds to Tf and enters the next cycle.

Pathologically, the abnormal accumulation of iron ions causes metabolic disorders, which is one of the characteristics of ferroptosis (47, 48). Neural damage caused by CIRI is closely associated with iron metabolism disorders (49). This imbalance of iron ions may be related to increased BBB permeability after CIRI. Under normal conditions, the presence of tight junctions between BBB endothelial cells strictly restricts the entry of blood-derived substances and cells into the brain (50–52). CIRI facilitates the release of matrix metalloproteinases from microglia, which disrupt BBB integrity (50, 53).

This allows circulating free iron to penetrate the brain parenchyma via the abnormally permeable barrier, resulting in pathological iron overload. In addition, IS induces dysfunction of iron export proteins, impeding iron efflux and causing its accumulation within cells. Therefore, regulating the expression levels of these proteins may be critical. Some studies have reported that serum Tf levels are markedly decreased in patients with IS (54, 55). Furthermore, another study found that reducing the expression of Tfr1 and divalent metal transporter 1 (DMT1) could alleviate CIRI and confer neuroprotective effects (56). Hepcidin, a small polypeptide hormone produced and released by the liver, is a central regulator of iron homeostasis and a significant contributor to iron overload in cerebral ischemia (37). Elevated hepcidin levels lead to the degradation of the iron export protein FPN1 and increase intracellular iron content in the central nervous system, which can trigger ferroptosis (57). The upregulation of hepcidin expression represents a novel mechanism underlying neuroinflammation and surgery-induced cognitive decline (58). A clinical study found that plasma hepcidin levels in patients with acute IS were significantly higher than those in healthy individuals (59). FPN1 is the only identified membrane transporter responsible for exporting iron from the cell and therefore plays a vital role in maintaining systemic iron homeostasis (60). In IS, upregulation of FPN1 can protect brain tissue against ischemic–hypoxic injury (61).

The imbalance of iron homeostasis results not only from abnormalities in iron import and export proteins but is also closely associated with dysregulation of intracellular iron storage proteins, particularly the ferritin degradation process. Ferritinophagy is a specialized cellular mechanism mediated by nuclear receptor coactivator 4 (NCOA4) that regulates iron homeostasis (62). Notably, NCOA4 and ferritin are key regulators of ferritinophagy (63). In IS, cerebral ischemia and hypoxia upregulate NCOA4 expression in neurons, and excessive ferritinophagy leads to ferritin degradation, thereby releasing large amounts of free iron. This excessive release disrupts iron homeostasis and ultimately triggers ferroptosis (64). This process directly links ferritinophagy to ferroptosis, and inhibition of ferritinophagy alleviates iron overload. Recently, Li et al. demonstrated that NCOA4 silencing reduced infarct volume and inhibited ferroptosis in tMCAO model rats (65). Furthermore, NCOA4 and FTH1 serve as critical targets linking ferroptosis and ferritinophagy after IS (66, 67). Blocking the interaction between NCOA4 and FTH1 inhibits ferritinophagy, thereby reducing intracellular Fe^2+^ content, effectively suppressing ferroptosis and alleviating brain injury in tMCAO rats (66). Consistently, Yang et al. reported that ginkgolide B inhibits ferroptosis and protects neurological function in tMCAO rats by interfering with the association between NCOA4 and FTH1 (68).

#### 2.2.2 Ischemic stroke and lipid peroxidation

Excessive iron ions can generate substantial amounts of ROS via the Fenton reaction (43, 69). These ROS can induce LPO and result in the accumulation of malondialdehyde (MDA) and lipid peroxides. This cascade reaction is a key inducer of ferroptosis and an important mechanism underlying tissue damage and organ failure (70). After cerebral ischemia–reperfusion, ROS levels are markedly elevated (71), which can subsequently stimulate the oxidation of polyunsaturated fatty acids (PUFAs) (72). As fatty acid side chains involved in the formation of cell membrane phospholipids, PUFAs readily interact with ROS, leading to membrane instability and increased permeability. These molecular events ultimately drive the LPO cascade reaction and ferroptosis. PUFAs, particularly arachidonic acid (AA) and adrenic acid (AdA), are first conjugated with coenzyme A (CoA) under the catalysis of ACSL4, generating AA/AdA-CoA derivatives. Subsequently, lysophosphatidylcholine acyltransferase 3 (LPCAT3) incorporates these derivatives into membrane phospholipids, especially phosphatidylethanolamine (PE), ultimately forming AA/AdA-PE (73, 74). Notably, AA/AdA-PE is the key phospholipid that induces ferroptosis (75). In the enzymatic pathway, lipoxygenases (LOX) oxidize AA/AdA-PE to produce phospholipid hydroperoxides (PL-OOH), such as AA/AdA-PE-OOH. These PL-OOH subsequently undergo β-cleavage to generate lipid peroxides, including MDA and 4-hydroxynonenal (4-HNE). Tuo et al. showed that ACSL4 overexpression induces brain tissue damage in tMCAO rats, whereas inhibition of ACSL4 activity through gene silencing prevents neuronal ferroptosis (76). Furthermore, Gong et al. found that inhibition of the ACSL4/LPCAT3 pathway reduces mitochondrial membrane potential and ROS levels and increases the formation of mitochondrial cristae, thereby alleviating iron accumulation and reducing lipid peroxide levels in the brain tissue of tMCAO rats (74). In the non-enzymatic pathway, Fe^2+^ reacts with hydrogen peroxide (H₂O₂) through the Fenton reaction to produce higher reactive hydroxyl radicals (·OH), and thereby triggers the LPO cascade reaction.

#### 2.2.3 Ischemic stroke and the antioxidant system

Glutathione peroxidase 4 (GPX4) is the only enzyme in humans that directly reduces lipid peroxides and is therefore regarded as a key target against ferroptosis. GPX4 converts glutathione (GSH) into glutathione disulfide and further reduces PL-OOH to phospholipid alcohols (PL-OH), thereby attenuating the toxicity of lipid peroxides (77–80). GSH, a key endogenous antioxidant, is synthesized under the regulation of the cystine metabolic pool, which is primarily mediated by the cystine/glutamate antiporter (system Xc^−^). This antiporter, an amino acid transport protein located on the cell membrane, consists of two proteins: solute carrier family 7 member 11 (SLC7A11) and solute carrier family 3 member 2 (SLC3A2). These proteins facilitate the exchange of extracellular cystine for intracellular glutamate across the plasma membrane (81–83). After entering the cell, cystine is reduced to cysteine, which is subsequently utilized for GSH synthesis. Some studies have indicated that upregulation of SLC7A11 can increase intracellular cysteine levels, thereby promoting GSH production and enhancing GPX4 activity, thus effectively inhibiting ferroptosis during cerebral ischemia (18, 83–85). Furthermore, Liu and colleagues demonstrated that serum levels of SLC7A11 and GPX4 are closely associated with disease severity in patients with acute IS. Lower serum levels of SLC7A11 and GPX4 were associated with more severe neurological impairment in these patients (86).

**Figure 1:**
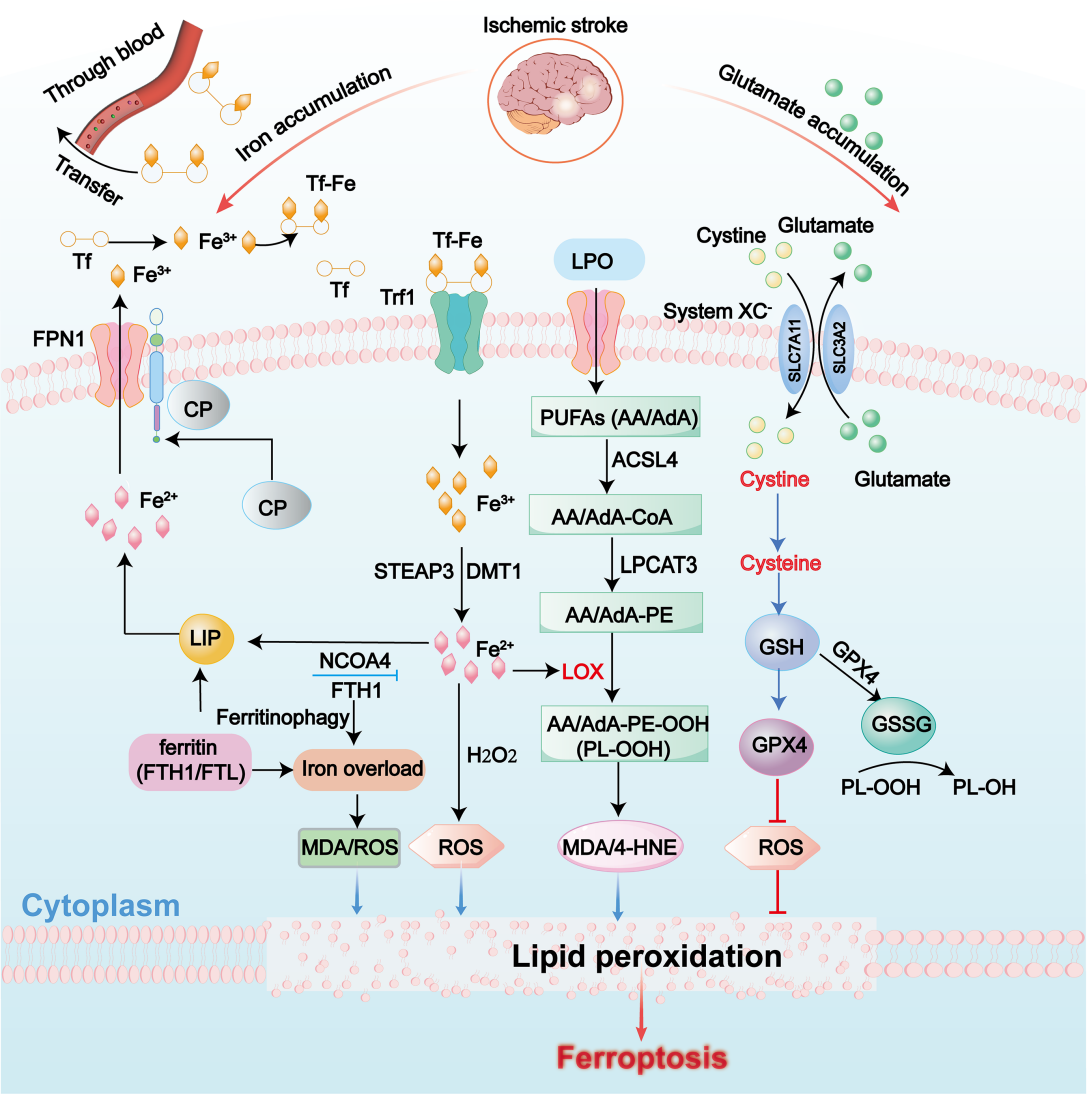
Mechanisms underlying the regulation of ferroptosis in ischemic stroke. LIP, labile iron pool; Tf, Transferrin; Tfr1, transferrin receptor 1; FPN1, ferroportin 1; DMT1, divalent metal transporter 1; STEAP3, six-transmembrane epithelial antigen of prostate 3; PUFAs, polyunsaturated fatty acids; PL-OOH, phospholipid hydroperoxide; GSSG, glutathione disulfide; GSH, glutathione; CP, ceruloplasmin; LOX, lipoxygenase; AA, arachidonic acid; AdA, adrenic acid. The brain tissue and ion channel graphics are sourced from the open SciDraw repository: Human Brain by Macauley Smith Breault, licensed under CC BY, https://doi.org/10.5281/zenodo.3925925; Ion channel by Marcelo Moglie, licensed under CC BY, https://doi.org/10.5281/zenodo.3926040.

Brain tissue is highly sensitive to oxidative stress due to its relatively weak endogenous antioxidant defense system. An imbalance in antioxidant capacity constitutes a primary cause of ferroptosis. Following IS, oxygen deprivation induces metabolic disturbances, impairs oxidative phosphorylation, and increases ROS production. This altered microenvironment disrupts the function of system Xc^−^ and inhibits GSH synthesis (87, 88). After ischemic injury, the accumulation of intracellular lipid peroxides reduces GPX4 activity, thereby increasing cellular oxidative stress. Inactivation or diminished activity of GPX4 prevents the decomposition of toxic PL-OOH, resulting in their intracellular accumulation and ultimately inducing ferroptosis in neurons (89). The regulatory mechanism of ferroptosis in IS is illustrated in Figure 1.

#### 2.2.4 Nrf2/HO-1 antioxidant pathway

Nuclear factor erythroid 2-related factor 2 (Nrf2) regulates ferroptosis and activates endogenous antioxidant programs to defend against oxidation (81, 90). In homeostasis, Kelch-like ECH-associated protein 1 (Keap1) negatively regulates Nrf2 in the cytoplasm, leading to its rapid degradation through the ubiquitin-proteasome pathway (90, 91). After cerebral ischemia, the resulting hypoxic microenvironment causes Nrf2 to be released from Keap1 and move into the nucleus. After entering the nucleus, Nrf2 activates a number of antioxidant factors and protects the system against oxidative damage (92). Nrf2 can inhibit ferroptosis by increasing antioxidant ability, probably due to its targeting of iron metabolism related proteins like FTH1/FTL (93). In fact, numerous proteins and enzymes inhibiting LPO and ferroptosis are regulated by Nrf2 (8). Recently, Zhang and colleagues demonstrated that Nrf2 activation can maintain oxidative balance and inhibit ferroptosis by reducing ROS levels and increasing GPX4 protein expression (94).

Heme oxygenase-1 (HO-1), a downstream target of Nrf2, is upregulated upon Nrf2 activation (95). HO-1 catalyzes heme degradation, converting free radicals into water and oxygen molecules, thereby reducing the accumulation of oxidative damage products. Beyond its antioxidant function, HO-1 can inhibit ferroptosis. This is mainly due to the metabolism of heme and the generation of its products biliverdin and bilirubin. These metabolites effectively reduce the levels of ROS and MDA (96–98). Recent studies have indicated that activation of the Nrf2/HO-1 signaling pathway can inhibit ferroptosis in neurons and protect brain tissue in tMCAO mice (99, 100). Paradoxically, heme metabolism also generates free iron, which promotes iron accumulation and can induce ferroptosis (101).

#### 2.2.5 FSP1/CoQ10 pathway

Ferroptosis suppressor protein 1 (FSP1) is a recently identified protein involved in redox regulation and biosynthesis pathways (102). As a ferroptosis inhibitor that operates independently and in parallel with GPX4, FSP1 suppresses ferroptosis by reducing coenzyme Q10 (CoQ10) in an NAD(P)H-dependent manner. The reduced form of CoQ10 acts as a lipophilic antioxidant capable of capturing free radicals, thereby blocking LPO (103, 104).

#### 2.2.6 DHODH/CoQH2 pathway

Dihydroorotate dehydrogenase (DHODH), a flavin-dependent enzyme located in the inner mitochondrial membrane, also participates in redox reactions with CoQ. DHODH catalyzes the conversion of dihydroorotate to orotic acid while reducing CoQ to dihydroquinol (CoQH2). CoQH2 functions as an antioxidant that captures free radicals, thereby inhibiting ferroptosis by reducing lipid peroxides (104). Notably, one study reported that the DHODH/CoQH2 axis can compensate for GPX4 deficiency. When GPX4 is acutely inactivated, DHODH-mediated CoQH2 production is markedly enhanced, thereby preventing ferroptosis within mitochondria (105).

#### 2.2.7 GCH1/BH4 pathway

The GTP cyclohydrolase 1 (GCH1)/tetrahydrobiopterin (BH4) axis represents another antioxidant and anti-ferroptosis pathway that operates independently of GPX4 and plays a critical role in vascular diseases (106). BH4, a key component of the antioxidant defense system, participates in various physiological processes, including nitric oxide (NO) biosynthesis and neurotransmitter regulation (107). GCH1 serves as the rate-limiting enzyme for BH4 synthesis, and BH4 functions in capturing free radicals (108). BH4 is readily oxidized to dihydrobiopterin (BH2), and through a redox cycle, it reduces endogenous oxidative free radicals, thereby inhibiting ferroptosis (109).

In the future, the DHODH/CoQH2, GCH1/BH4, and FSP1/CoQ10 axes represent promising novel regulatory pathways for anti-ferroptosis and antioxidant defense in IS, meriting further in-depth investigation.

## 3 HIF-1α

HIF-1 was first discovered by Semenza and colleagues during studies of the EPO gene (110). HIF-1 is composed of HIF-1α (120 kDa) and HIF-1β (91 kDa), both belonging to the basic helix–loop–helix transcription factor family (111). HIF-1α functions as the central regulator of oxygen homeostasis, with its activity highly sensitive to oxygen levels (112, 113). Under normoxic conditions, HIF-1α is regulated by prolyl hydroxylase domain (PHD) enzymes and factor-inhibiting HIF-1 (FIH-1) (114). PHD hydroxylates HIF-1α, which is subsequently ubiquitinated by an E3 ubiquitin ligase complex and degraded via the 26S proteasome (115–117). Concurrently, FIH-1 inhibits HIF-1α activity by blocking its interaction with the transcriptional coactivators p300/CBP (CREB-binding protein) (118). Under hypoxic or ischemic stress, the hydroxylation activity of PHD and FIH-1 is reduced, resulting in rapid stabilization and activation of HIF-1α, thereby enabling it to regulate the cellular adaptive response to hypoxia (119–121). One study demonstrated that PHD knockout enhanced HIF-1α stability and the expression of critical target genes under low oxygen conditions (122). However, HIF-1α activation exhibits a dual role in IS by mediating both protective and destructive effects on brain tissue. The mechanisms underlying this duality involve broader HIF-1α-mediated pathways in IS, including angiogenesis, glucose metabolism, inflammation, BBB disruption, apoptosis, and autophagy (29, 33, 123–128), which are recognized as general regulatory processes.

### 3.1 The neuroprotective effects of HIF-1α in ischemic stroke

HIF-1α activation exerts a neuroprotective effect in cerebral ischemia primarily by promoting angiogenesis, exerting antioxidant effects, improving glucose metabolism, and regulating autophagy.

As a classic downstream target gene of HIF-1α, VEGF is an important regulatory factor for neuroprotection and neuroregeneration in ischemic brain tissue (128). It is a vascular permeability factor associated with endothelial cell differentiation, proliferation, and migration and also serves as a key signal for inducing angiogenesis (129). Under ischemic and hypoxic conditions, HIF-1α is rapidly activated and binds to the hypoxia response element in the VEGF promoter, thereby increasing its transcription (130, 131). After IS, HIF-1α-mediated upregulation of VEGF enhances angiogenesis and improves neurological recovery and is considered a driver of angiogenesis (132–137). Another angiogenic pathway involves the Notch signaling pathway, which regulates cellular adaptation to hypoxia. One study indicated that the HIF-1/VEGF/Notch signaling pathway plays a synergistic role in driving hypoxia-induced angiogenesis (135). Xu et al. removed the nylon filament after 1 h of cerebral ischemia, and the rats were sacrificed after 24 h of reperfusion. The results showed that HIF-1α inhibitors reduced the levels of the angiogenic proteins VEGF and Notch1 and aggravated brain injury in tMCAO rats (138).

HIF-1α also mediates neuroprotection by increasing EPO expression, which can inhibit neuronal apoptosis and restore neuronal function. Recent studies have shown that the HIF-1α/EPO axis reduces cerebral infarct volume, enhances oxygen delivery and improves brain injury (24, 139). Zhang et al. sacrificed rats subjected to 90 min of tMCAO followed by 24 h of reperfusion and observed that elevated HIF-1α and EPO levels effectively alleviated brain injury on the ischemic hemisphere (140). Upregulation of EPO transcription further increases cerebral blood flow by promoting angiogenesis after cerebral ischemia (141).

During IS, the rapid reduction of cerebral blood flow leads to decreased oxygen and glucose levels, which can impair mitochondrial function and disrupt energy metabolism (142). HIF-1α enhances cell viability by increasing glucose uptake capacity and transporter activity, promoting glycolysis, and maintaining redox homeostasis. Glucose transport into the brain is primarily mediated by glucose transporters, mainly glucose transporter 1 (GLUT1) and GLUT3. GLUT1 facilitates glucose delivery to brain tissue, whereas GLUT3, characterized by high glucose affinity, ensures neuronal glucose uptake (143). One study found that HIF-1α upregulates GLUT1 and GLUT3, enabling neurons to exhibit enhanced glucose uptake capacity under hypoxic conditions (144). In cell models of oxygen–glucose deprivation/reoxygenation (OGD/R), HIF-1α knockout disrupts key components of cellular redox balance and suppresses the pentose phosphate pathway as well as the expression of transporters such as GLUT1 (25).

Recently, one study has also demonstrated that HIF-1α reduces ischemic infarct volume in tMCAO models by regulating autophagy-related proteins, including LC3 and P62 (127). Furthermore, HIF-1α expression is positively correlated with the Nrf2/HO-1 pathway (145), and activation of HIF-1α upregulates Nrf2/HO-1 levels, thereby effectively alleviating motor dysfunction in a mouse model of IS induced by endothelin-1 (ET-1) injection (146). Conversely, loss of HIF-1α function exacerbates ischemic injury. Nashwa et al. reported that in a mouse model of IS induced by ET-1 injection, HIF-1α knockout aggravated motor dysfunction, decreased the number of neurons in the cerebral cortex following ischemia, and increased the activity of microglia and astrocytes (147). Zhang et al. found that in mice subjected to 1 h ischemia followed by 72 h reperfusion, HIF-1α knockdown significantly worsened motor dysfunction and increased neuronal apoptosis and oxidative stress at 72 h after reperfusion (148).

### 3.2 The effects of neurological damage on HIF-1α in ischemic stroke

The effects of activated HIF-1α are not limited to protection but also include detrimental outcomes in IS. In contrast to the above findings, one study reported that HIF-1α knockout attenuated tMCAO-induced brain injury and reduced BBB permeability (149). Helton et al. used HIF-1α-deficient mice to establish a bilateral common carotid artery occlusion (BCCAO) model simulating IS injury and observed that HIF-1α deficiency significantly reduced neuronal apoptosis in the hippocampus and cerebral cortex, accompanied by downregulation of multiple pro-apoptotic genes (150).

This adverse effect is likely closely associated with HIF-1α involvement in inflammation and oxidative stress. Chen and colleagues recently demonstrated that HIF-1α knockdown decreased neuroinflammation after 2 h of ischemia and 24 h of reperfusion in tMCAO model rats via reducing the CXCR4/NF-κB pathway (33). In addition, Jiang et al. reported significant upregulation of HIF-1α and the NLRP3 inflammasome in the infarcted cortex following 2 h of ischemia and 24 h of reperfusion in tMCAO model rats, while HIF-1α inhibition markedly decreased NLRP3 levels (32).

Another mechanism involves the interaction between HIF-1α and oxidative stress. Under hypoxic conditions, NO acts as a key factor in maintaining HIF-1α activation. NO accumulation can induce HIF-1α overexpression, which subsequently upregulates pro-inflammatory cytokines including TNF-α, IL-1β, and NF-κB p65, thereby promoting neuronal apoptosis (151). Li et al. reported that isoflurane postconditioning activates HIF-1α in tMCAO model rats, leading to upregulation of inducible nitric oxide synthase (iNOS) expression, whereas prolonged exposure to high concentrations of isoflurane results in excessive HIF-1α-driven iNOS activation and NO production, leading to consequent neurotoxicity (152).

**Figure 2:**
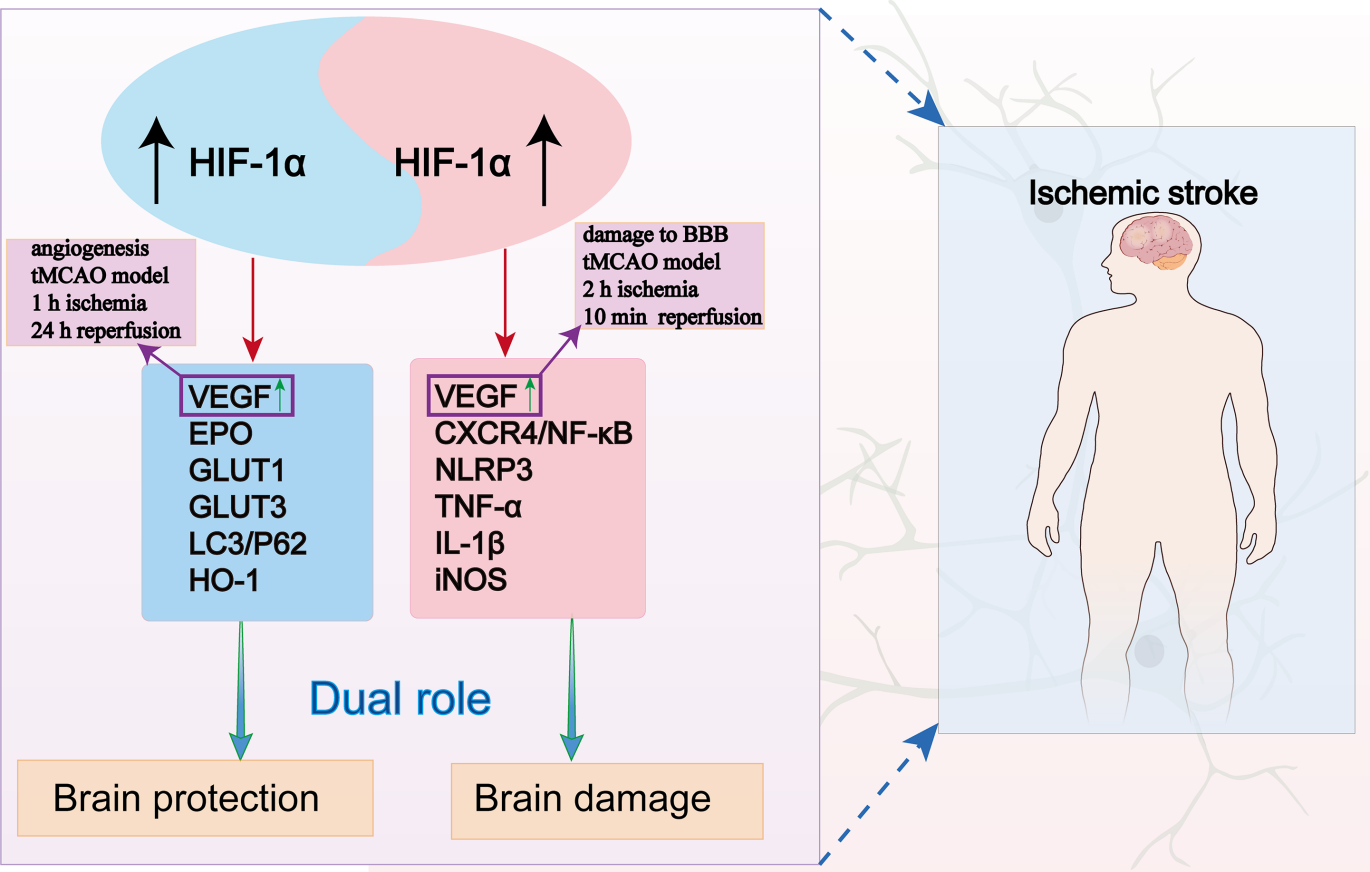
Mechanisms underlying the dual neuroprotective and injurious roles of HIF-1α in ischemic stroke. The neuron and human body graphics are obtained from SciDraw and NIH BioArt respectively: pyramidal neuron by Federico Claudi, licensed under CC BY, https://doi.org/10.5281/zenodo.3925905; Human Anatomy by Ryan Kissinger, in the Public Domain, https://bioart.niaid.nih.gov/bioart/519.

Furthermore, HIF-1α can exacerbate IS injury by increasing BBB permeability. This occurs because HIF-1α disrupts BBB integrity through upregulation of matrix metalloproteinase-2 (MMP-2) and VEGF, leading to decreased levels of capillary tight junction proteins (153, 154). One study reported that specific deletion of HIF-1α reduced BBB leakage and decreased cerebral infarct volume in tMCAO rats (149). Notably, the detrimental effect of HIF-1α is closely related to the duration of ischemia. Some studies have shown that 1 h after cerebral ischemia, HIF-1α increases BBB permeability via VEGF upregulation and promotes necroptosis through the Notch pathway (29, 155, 156). Another study demonstrated that in tMCAO model rats subjected to 2 h of ischemia followed by 10 min of reperfusion, HIF-1α was significantly activated in neurons, which subsequently promoted VEGF and MMP-2 secretion, resulting in BBB damage. In contrast, no change in HIF-1α was observed in astrocytes (157). This discrepancy is likely due to the absence of PHD enzyme expression in astrocytes, whereas HIF-1α upregulation in neurons is highly dependent on PHD enzymes. Consequently, astrocytes are more effective in inducing ischemic tolerance (158). Blocking VEGF in the early stages of stroke can reduce BBB permeability and lower the risk of hemorrhagic transformation after IS (29). Collectively, these findings suggest that while HIF-1α promotes vascular regeneration via VEGF activation, its early induction during IS may damage the brain by disrupting the BBB. Therefore, suppressing HIF-1α in the initial phase of IS may confer neuroprotection (Figure 2).

### 3.3 Dynamic regulation and dual roles of HIF-1α in ischemic stroke

Previous studies have confirmed that the dual function of HIF-1α in IS depends on both the duration and severity of the ischemic insult (159–161). HIF-1α levels change dynamically with ischemia duration during IS. For instance, in a mouse tMCAO model, HIF-1α mRNA in the infarct area reached its maximum level at 24 h post-ischemia and declined thereafter (162, 163). Another study indicated that HIF-1α levels in the vascular endothelium of tMCAO model mice peaked 6 h after reperfusion and remained elevated through 24 h (150). Additionally, Zhang et al. reported that in the ischemic cortex of MCAO rats, HIF-1α expression began to increase 1 h post-reperfusion, peaked at 12 h, and then declined by 24 h (164). Similarly, Mu et al. observed in tMCAO mice that cortical HIF-1α levels peaked within 8 h and gradually declined after 24 h (165). These findings suggest that HIF-1α levels change dynamically during IS, rising rapidly within the first 24 h and then decreasing gradually, and that this variation may be critical to its dual role.

Barteczek et al. found that double silencing of HIF-1α and HIF-2α in tMCAO mice markedly reduced neuronal death and brain edema after 24 h of reperfusion. However, after 72 h of reperfusion, neuronal function was significantly impaired, with increased apoptosis and decreased angiogenesis (22). Another study revealed that HIF-1α knockout at 0.5 h post-ischemia reduced brain edema and apoptosis, whereas HIF-1α knockout at 8 h exacerbated neurological injury and decreased VEGF expression (31). In contrast, HIF-1α activation may exert neuroprotective effects in IS at time points beyond 24 h (159). Amin et al. found that increased HIF-1α expression at 12 days post-stroke in a mouse model of IS induced by ET-1 injection downregulated pro-inflammatory cytokines, including iNOS, NF-κB, and TNF-α, resulting in improved neurological function (166). Another study also reported that elevated HIF-1α levels at 28 days in a tMCAO model rat protected rat brain tissue (167). Collectively, these findings indicate that inhibition of HIF-1α is protective in the early phase of IS (<24 h), whereas as ischemia progresses and HIF-1α levels gradually decline, its inhibition becomes detrimental.

**Figure 3:**
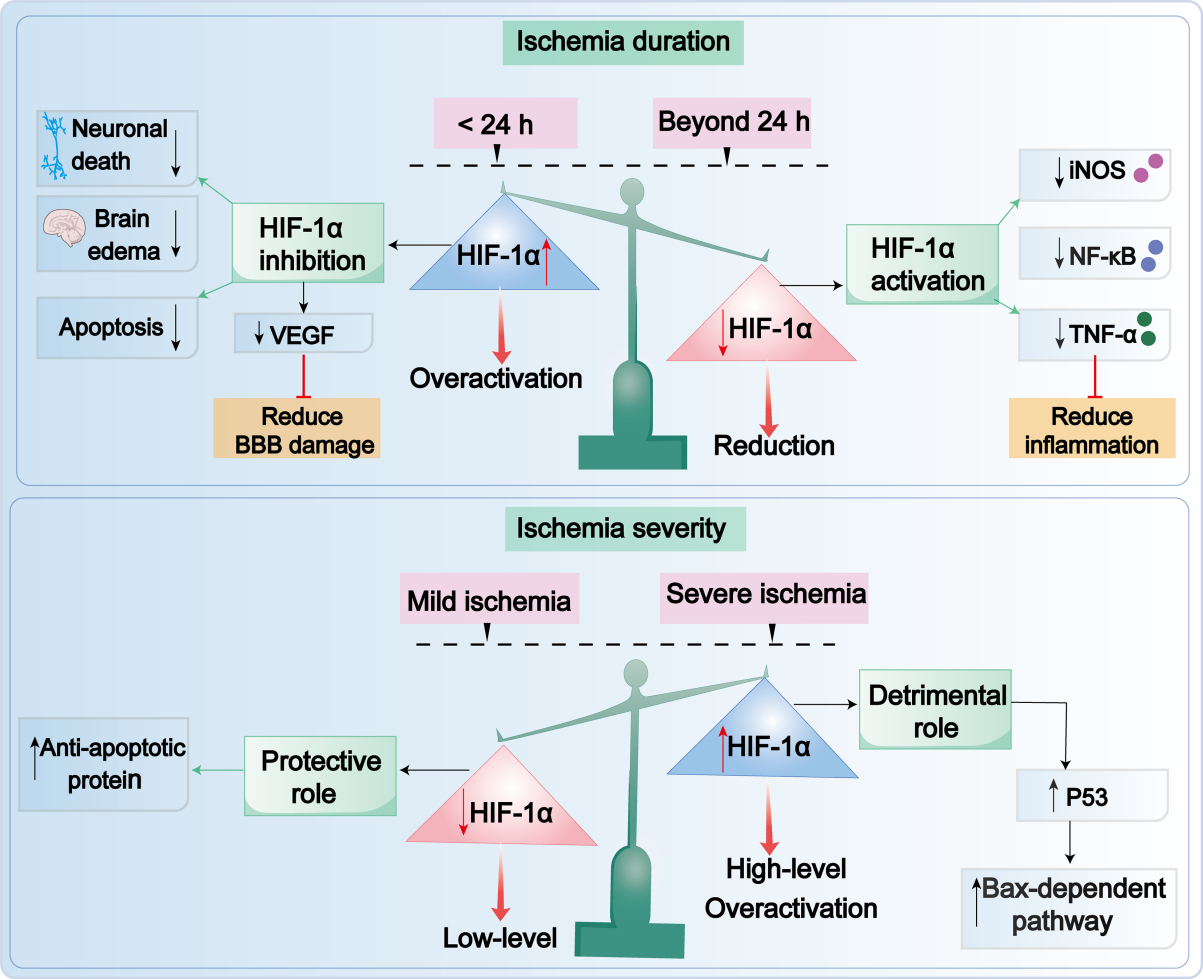
Changes in HIF-1α levels regulate the outcome of ischemic stroke based on the duration and severity of ischemia. The neuron and brain tissue graphics are obtained from SciDraw and NIH BioArt, respectively: Human Brain by Macauley Smith Breault, licensed under CC BY, https://doi.org/10.5281/zenodo.3925925; Human Anatomy by Ryan Kissinger, in the Public Domain, https://bioart.niaid.nih.gov/bioart/519.

The protective or damaging effects of HIF-1α are also closely related to the severity of cerebral ischemia (168, 169). In mild hypoxia, HIF-1α exerts a protective function through the induction of anti-apoptotic proteins. However, under severe hypoxia, HIF-1α can bind to p53. This interaction induces mitochondrial cytochrome c release via a Bax-dependent mechanism and caspase activation, thereby promoting neuronal injury (170, 171). A prospective study measured serum HIF-1α concentrations in patients with acute IS at 24, 48, 72, and 96 h. Correlation analysis combining infarct volume and National Institutes of Health Stroke Scale scores showed that higher HIF-1α levels were significantly associated with greater stroke severity (113). Another clinical study also demonstrated that serum HIF-1α levels were significantly elevated in patients with IS compared with healthy controls, and that higher levels were significantly correlated with poorer survival outcomes (172). In addition, Li et al. reported that serum HIF-1α levels were significantly higher in patients with severe and moderate cerebral infarction than in those with mild cerebral infarction (168). These findings suggest that HIF-1α may become overactivated following severe ischemic–hypoxic injury, thereby shifting from a protective to a detrimental role (Figure 3).

In summary, HIF-1α exerts either neuroprotective or detrimental effects after IS by regulating multiple downstream pathways, with its dual role critically dependent on the duration and severity of ischemia, cell type, and model differences. The above studies indicate that HIF-1α confers protection via the VEGF/Notch1 pathway in tMCAO models subjected to 1 h of ischemia (138), whereas in models with 2 h of ischemia, HIF-1α is significantly upregulated alongside the NLRP3 inflammasome, thereby promoting injury (32). Clinical studies suggest that more severe neurological damage is associated with higher levels of HIF-1α (113), indicating that HIF-1α may be overactivated in patients with severe IS, which may be a key reason for its shift from protection to injury. Furthermore, HIF-1α levels also differ among brain cell types (157). Model differences also warrant attention, as IS models such as tMCAO, BCCAO, ET-1 and OGD differ in ischemic modality, injury scope, severity, and cellular microenvironment (173), all of which can influence HIF-1α stability and expression levels, thereby regulating downstream signaling pathways.

## 4 HIF-1α regulation of ferroptosis in ischemic stroke

The above studies indicate that HIF-1α exerts both protective and injurious effects in IS by regulating multiple downstream pathways, which represent general pathological mechanisms mediated by HIF-1α. Recent studies have further shown that HIF-1α also plays an important role in regulating ferroptosis (174, 175). The following section will focus on how HIF-1α exerts its dual regulatory function on ferroptosis in IS by modulating ferroptosis-specific mechanisms, including iron metabolism, lipid metabolism, and oxidative stress pathways.

### 4.1 HIF-1α and iron metabolism

HIF-1α can induce several iron homeostasis proteins, including DMT1, Tfr1, FPN1, and hepcidin (176, 177). Yao et al. demonstrated that elevated HIF-1α levels increased FPN1 expression in the hippocampal neurons of rats at 24 h after tMCAO and inhibited ferroptosis through activation of the Nrf2/HO-1 signaling pathway (178). Similarly, another study reported that activation of HIF-1α improved iron utilization in the blood by suppressing hepcidin and increasing FPN1 levels (179). In the distal MCAO (dMCAO) mouse model, hepcidin knockdown in the ischemic cortex at 28 days post-ischemia was found to downregulate PHD2 and upregulate HIF-1α expression, thereby initiating endogenous repair processes and promoting functional recovery after ischemic injury (174). These studies indicate that upregulation of HIF-1α inhibits ferroptosis by modulating iron metabolism-related proteins in IS at time points beyond 24 h.

Conversely, HIF-1α can also promote ferroptosis through the regulation of iron metabolism. One study found that HIF-1α activation upregulated Tfr1 expression, promoted intracellular iron accumulation, and then exacerbated oxidative damage and LPO responses (180). Ding et al. reported that elevated HIF-1α in the cerebral cortex, hippocampus, and striatum after 24 h of reperfusion in tMCAO model rats upregulated Tfr1 and hepcidin expression, resulting in cerebral iron overload (37). Notably, although Ding et al. (37) and Yao et al. (178) used the same model and reperfusion time, their results were opposite, possibly due to individual animal differences and variations in injury severity in the IS model.

### 4.2 HIF-1α and lipid metabolism

ROS serve as a critical initiator of the LPO cascade, with HIF-1α expression showing a significant positive correlation with ROS levels. Under hypoxic conditions, substantial ROS production stabilizes the HIF-1α protein, thereby enhancing its activity (4, 181–183). One study found that HIF-1α knockdown reduced infarct volume and ROS generation after 2 h of cerebral ischemia followed by 24 h of reperfusion in tMCAO rats (33). These findings support a positive regulatory relationship between HIF-1α and ROS levels.

In contrast, Wu et al. reported in an atherosclerosis model that activation of the HIF-1 signaling pathway improved mitochondrial function, reduced ROS levels, and inhibited both ferroptosis and apoptosis (184). Furthermore, in neonatal rats with hypoxic–ischemic brain injury, the expression of the ferroptosis-related transcription factor HIF-1α was markedly reduced, whereas brain tissue exhibited a significant increase in ROS and 4-HNE (185). Although these studies suggest a negative regulatory role of HIF-1α on ROS expression, they provide only indirect evidence that is inconsistent with observations in IS. Therefore, further studies in IS models are required to clarify the regulatory interaction between HIF-1α and ROS.

As vital components of brain tissue, lipids become increasingly susceptible to peroxidation when ACSL4 expression is elevated. ACSL4 is a key isoenzyme involved in PUFA metabolism. Its overexpression exacerbates ischemic brain injury, enlarges the infarct area, and accelerates neuronal ferroptosis. Interestingly, recent studies have shown that HIF-1α can inhibit ACSL4 transcription by binding to its promoter region (34, 35). Additionally, Cui et al. reported that in human neuroblastoma cells subjected to 2 h of OGD, HIF-1α activation suppresses ferroptosis through downregulation of ACSL4 expression (36). However, Jin and colleagues found that in HT22 cells subjected to 4 h of OGD, the STAT3/HIF-1α/PTRF axis promotes the accumulation of LPO products such as MDA and ROS by upregulating PLA2G4A expression, ultimately inducing ferroptosis (186).

As a classic *in vitro* model of IS, the OGD model exhibits a positive correlation between the degree of injury and OGD duration: both 2 h and 4 h of OGD can induce ischemic injury, but the injury induced by 4 h is more severe. Short-duration OGD (2 h) induces moderate activation of HIF-1α, which inhibits ferroptosis by downregulating ACSL4. In contrast, long-duration OGD (4 h) causes excessive activation of HIF-1α, shifting its regulatory effect and promoting ferroptosis. This difference may explain why two studies using the same model obtained opposing results.

### 4.3 HIF-1α and oxidative stress

System Xc^−^ is a key transporter responsible for cellular cystine uptake and glutamate efflux. Notably, cystine is essential for the synthesis of GSH and GPX4. In models of cerebral hypoxic ischemia, HIF-1α can stimulate GSH synthesis by upregulating the glutamate-cysteine ligase modifier subunit (187). Furthermore, HIF-1α regulates downstream targets such as SLC7A11 and GPX4. In a rat model of peripheral neuronal injury, activation of HIF-1α upregulated the expression of SLC7A11 and GPX4 (188). Similarly, in a mouse model of renal ischemia–reperfusion injury, HIF-1α activation upregulated SLC7A11 and GPX4 expression in kidney tissue, thereby inhibiting ferroptosis and alleviating acute kidney injury (189). However, these studies provide only indirect evidence. Recently, Wang et al., using a tMCAO *in vivo* model (1 h ischemia and 24 h reperfusion) and an OGD/R *in vitro* model, demonstrated that elevated HIF-1α levels promote SLC7A11 expression, resulting in upregulation of GPX4 and thereby alleviating brain endothelial cell injury induced by ferroptosis (190).

**Figure 4:**
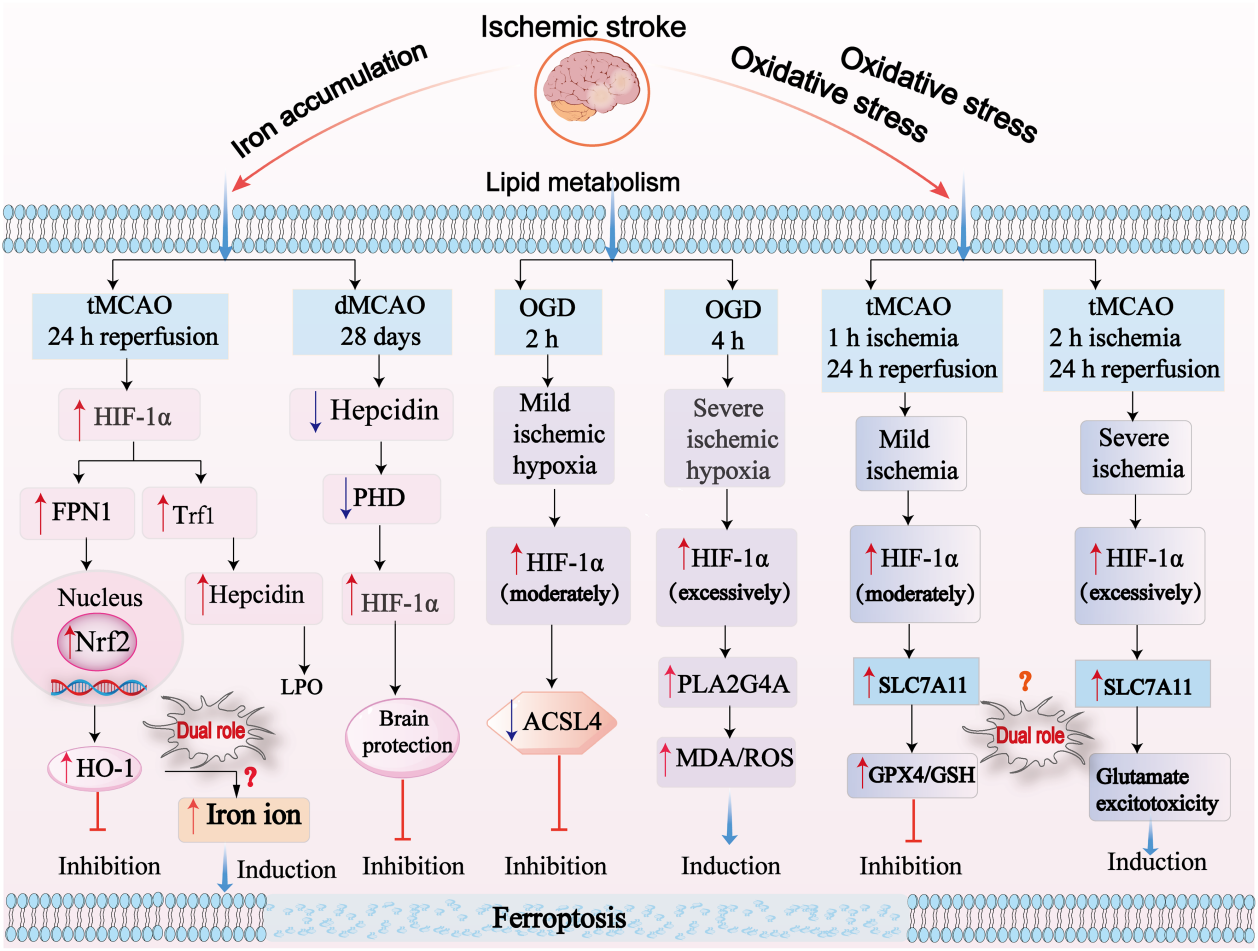
Regulatory mechanism of HIF-1α in ferroptosis induction and suppression. The roles of the HIF-1α/SLC7A11 and HIF-1α/HO-1 signaling axes in ferroptosis remain controversial, which requires further *in vivo* verification. The brain tissue graphic is sourced from the open SciDraw repository: Human Brain by Macauley Smith Breault, licensed under CC BY, https://doi.org/10.5281/zenodo.3925925.

In contrast, Ma et al. found that HIF-1α was the main mediator of ferroptosis induced by the OGD/R model (4 h of OGD followed by 24 h of reoxygenation). Downregulation of HIF-1α improved cell viability, significantly reduced intracellular Fe^2+^ and MDA levels, and increased GSH content (191). Additionally, HIF-1α serves as a critical trigger for the disruption of extracellular glutamate homeostasis, and it can directly bind to the SLC7A11 (xCT) promoter region. One study indicated that in a tMCAO model (2 h ischemia and 24 h reperfusion), HIF-1α activation promotes SLC7A11 (xCT) expression. This protein facilitates glutamate release and excitotoxicity, whereas conditional knockout of HIF-1α suppresses extracellular glutamate levels and N-methyl-D-aspartate receptor activation in ischemic brain tissue (192). Similarly, Zhong et al. also reported that inhibition of the HIF-1α/SLC7A11 axis and mitochondrial activity alleviated ferroptosis in tMCAO model rats (2 h ischemia and 48 h reperfusion) (193). According to current research, whether the HIF-1α/SLC7A11 axis exerts an inhibitory or promotive effect on ferroptosis in IS remains controversial (Figure 4).

Collectively, the degree of injury differs between tMCAO models subjected to 1 h and 2 h of ischemia. Compared with 2 h of ischemia, the 1 h of ischemia model induces milder injury, and moderate activation of HIF-1α may exert an anti-ferroptosis effect. However, as ischemia time prolongs, the degree of injury worsens, and HIF-1α becomes overactivated, which may be the reason for the shift of SLC7A11 from anti-ferroptosis to pro-ferroptosis.

### 4.4 Regulation of ferroptosis by the HIF-1α-related pathway

#### 4.4.1 HIF-1α/HO-1 pathway

Under hypoxic conditions, elevated HIF-1α upregulates its downstream target gene HO-1. Paradoxically, HO-1 activation can promote ferroptosis via the release of Fe^2+^ (48). Treatment with an HIF-1α stabilizer further increased HO-1 and ACSL4 mRNA expression while downregulating GPX4 expression, suggesting that activation of HIF-1α and HO-1 promotes ferroptosis (194). Recently, Shang et al. used a permanent MCAO rat model and sacrificed animals after 12 h. They found that inhibition of the HIF-1α/HO-1 pathway with YC-1 significantly reduced LPO, ROS, and Fe^2+^ levels, downregulated iron metabolism-related proteins Tf, and upregulated FTH1 expression, thereby inhibiting ferroptosis (195). In contrast, several studies have indicated that activation of the Nrf2/HO-1 pathway alleviates ferroptosis and ameliorates brain injury in tMCAO mice (99, 100).

This dual role is closely related to HO-1 function. HO-1 catalyzes heme degradation to generate biliverdin and bilirubin, which possess anti-inflammatory and antioxidant effects. Studies have reported that bilirubin is an effective ROS scavenger (96, 97). On the other hand, HO-1 releases free iron during heme degradation, creating conditions conducive to ferroptosis (196). Notably, the pro-ferroptotic role may be critically dependent on excessive HO-1 activation, as excessive heme degradation can lead to iron accumulation (197). Suttner et al. confirmed that iron released from heme degradation exacerbates oxidative injury only when HO-1 is overexpressed beyond a specific threshold, whereas low-to-moderate HO-1 expression predominantly exerts cytoprotective effects (198).

Notably, the dual role of HO-1 is also associated with the expression levels of iron metabolism-related proteins such as ferritin and FPN1. The protective effect of HO-1 is attributed to increased ferritin expression and its iron-chelating function, and free iron released during heme catabolism induces ferritin expression (199). FTH1, the ferroxidase subunit of ferritin, can store excess iron when upregulated, thereby enhancing cellular antioxidant defense and protection. However, when HO-1 is overexpressed, iron released from heme degradation accumulates and induces LPO and ferroptosis. Furthermore, HO-1 upregulates FPN1 expression to promote iron export, and increased FPN1 expression effectively protects brain tissue from ischemic and hypoxic injury (61).

#### 4.4.2 HIF-1α/YTHDF1/BECN1 pathway

YTHDF1, the most potent m6A reader protein among the five human YTH domain-containing family members, regulates target gene expression (200). In hepatocellular carcinoma, HIF-1α upregulates YTHDF1, which is functionally linked to ferroptosis-related genes (201). Recently, Ma et al. discovered that in an OGD/R neuronal model, HIF-1α can bind to the promoter region of YTHDF1 and promote its transcription. Moreover, they found that YTHDF1 induces ferroptosis by enhancing the stability of BECN1 mRNA (191). This finding suggests that the HIF-1α/YTHDF1/BECN1 axis plays a significant role in promoting ferroptosis during IS (Table 1).

**Table 1:** The regulatory effect of activating or inhibiting HIF-1α expression on ferroptosis in ischemic stroke.

HIF-1α status	Species	Observation location	Model type	Timing of ischemia/reperfusion	Function	Outcome	Administration window	References
Elevated HIF-1α levels	Sprague–Dawley rats	Hippocampal neurons	tMCAO model	24 h after reperfusion	FPN1↑, Nrf2/HO-1 expression↑	Inhibit ferroptosis	Pretreatment	(178)
Elevated HIF-1α levels	Wistar rats and BALB/c mice	Ischemic cerebral cortex/hippocampus/striatum	tMCAO model	Ischemia for 1 h and 24 h of reperfusion	Tfr1↑, hepcidin↑, iron↑,	Induce ferroptosis	Pretreatment	(37)
Activation of HIF-1α	Human neuroblastoma cell line	Not applicable	OGD model	Subjected to OGD for 2 h	ACSL4 expression↓	Inhibit ferroptosis	Pretreatment/post-ischemia	(36)
Activation of HIF-1α	HT22 cells	Not applicable	OGD/R model	4 h of OGD followed by 8 h of reoxygenation	MDA↑, ROS↑, PLA2G4A↑	Induce ferroptosis	Post-ischemia	(186)
Elevated HIF-1α levels	Human brain microvascular endothelial cell/ C57BL/6 mouse	Endothelial cell	OGD/R model, tMCAO model	2 h of OGD followed by 24 h of reoxygenation /ischemia for 1 h and 24 h of reperfusion	SLC7A11↑, GPX4↑,	Inhibit ferroptosis	Post-ischemia	(190)
Elevated HIF-1α levels	HT-22 cells	Not applicable	OGD/R model	4 h of OGD followed by 24 h of reoxygenation	GSH↓, Fe^2+^↑, MDA↑, YTHDF1↑, BECN1↑	Induce ferroptosis	Pretreatment	(191)
Activation of HIF-1α	C57BL/6J mice/ Sprague–Dawley rats	Ischemic cerebral cortex	tMCAO model	Ischemia for 2 h and 24 h of reperfusion	SLC7A11↑, N-methyl-D-aspartate receptor↑	Induce ferroptosis	Pretreatment	(192)
Inhibition of HIF-1α	Sprague–Dawley rats	Penumbral brain tissue	tMCAO model	Ischemia for 2 h and 48 h of reperfusion	GSH↑, Fe^2+^↓, MDA↓, SLC7A11↓, glutamate↓	Inhibit ferroptosis	Post-ischemia	(193)
Inhibition of HIF-1α	Sprague–Dawley rats	Ischemic hemisphere	tMCAO model	Ischemia for 2 h and 24 h of reperfusion	Infarct volume↓, ROS↓, MDA ↓	Inhibit ferroptosis	Pretreatment	(33)

#### 4.4.3 HIF-1α/VEGF pathway

As previously described, activated HIF-1α promotes post-ischemic angiogenesis by upregulating VEGF, thereby improving neurological function. However, the HIF-1α/VEGF axis extends beyond its pro-angiogenic role, also alleviating hypoxic–ischemic brain injury by inhibiting ferroptosis. Recent evidence confirmed that activation of the HIF-1α/VEGFA pathway significantly reduces ACSL4 levels, thereby suppressing ferroptosis and mitigating hypoxic–ischemic brain injury (202).

## 5 Treatment prospects

### 5.1 PHD inhibitor

PHD inhibitors, as activators of HIF-1α, stabilize HIF-1α by inhibiting PHD activity, thereby upregulating the transcription of downstream target genes such as VEGF and EPO. In mice with chronic cerebral hypoperfusion, PHD inhibition improves cognitive function (147). PHD inhibitors exert neuroprotective effects by suppressing ferroptosis, thereby ameliorating neurodegenerative diseases (203).

Several PHD inhibitors are currently under investigation and development. Roxadustat (FG-4592) is a potent PHD inhibitor with greater specificity for HIF-1α than dimethyloxalylglycine (DMOG). This specificity may reduce the iron metabolism disturbances caused by broader-spectrum PHD inhibitors, potentially providing a promising hypoxia-mimetic agent for patients. At present, FG-4592 is being studied for anemia in patients with chronic kidney disease and has advanced to the clinical trial stage (204). Furthermore, Reischl and colleagues found that another PHD inhibitor, FG-4497, can activate HIF-1α and exert neuroprotective and vascular leakage-reducing effects in tMCAO model mice (1 h ischemia and 24 h reperfusion) (205).

Notably, DMOG serves as a widely used cell-permeable PHD inhibitor. By inhibiting PHD enzyme activity, DMOG stabilizes and accumulates HIF-1α, thereby activating downstream hypoxic response pathways (206) and regulating cellular adaptation and survival under hypoxic conditions (207, 208). In a rat model of tMCAO (90 min of ischemia followed by 48 h of reperfusion), Yang et al. administered DMOG via intraperitoneal injection at 60 min after reperfusion. The results showed that this treatment significantly reduced cerebral infarct volume, improved neurological function, and upregulated anti-inflammatory cytokines such as interleukin-4 and interleukin-10 in both peripheral blood and ischemic brain tissue (209). Furthermore, in a mouse model of cerebral ischemia, Amin et al. administered DMOG via daily intraperitoneal injection starting at 60 min after ischemia induction for 10 consecutive days. The results demonstrated that DMOG significantly upregulated HIF-1α, VEGF, Nrf2, and HO-1 expression in the ischemic hemisphere, improved neurological deficits and motor coordination, and promoted angiogenesis (146). Recently, Chen et al. demonstrated that DMOG treatment could suppress p53, increase SLC7A11 and GPX4 expression, and thereby inhibit ferroptosis in dopaminergic neuron-like cells (210).

Although PHD inhibitors exhibit neuroprotective potential, several concerns remain. First, the timing of PHD inhibitor administration is critical. In the early stage of IS, within hours after reperfusion, HIF-1α activation may exacerbate BBB leakage and injury (29). Accordingly, the therapeutic time window for PHD inhibitors in IS requires further investigation, as early administration may worsen outcomes. While PHD inhibitors such as FG-4592 have advanced to clinical trials for renal anemia, their efficacy and safety in patients with IS remain unverified. Most current studies are limited to preclinical animal models, and evidence from large-scale, randomized, double-blind clinical trials is lacking. Future research should focus on optimizing the administration time window of PHD inhibitors to evaluate their potential benefits and risks in patients with IS.

### 5.2 Iron chelator

Deferoxamine (DFO) is the most extensively studied iron chelator. It indirectly activates HIF-1α by chelating iron ions and is also a classic inhibitor of ferroptosis (211). DFO has shown therapeutic potential in various neurological disorders such as Alzheimer’s disease, Parkinson’s disease, and IS (212). Although some studies suggest harmful roles of HIF-1α in IS, it can be neuroprotective against post-ischemic ferroptosis (213, 214). For instance, Ma et al. found that DFO treatment reduced cerebral infarct volume and improved neurological deficit scores in MCAO models. This protective effect was associated with inhibition of ferroptosis, mediated by elevated GPX4 levels (214). Given its high iron affinity, the FDA has approved DFO for the treatment of iron overload (215). Preclinical studies have shown that systemic administration of DFO may prevent and treat IS. However, there are several limitations to its clinical application. First, DFO is rapidly metabolized by plasma globulins, resulting in a plasma half-life of approximately 30 min. This requires frequent administration, which compromises patient compliance. Second, DFO has dose- and time-dependent toxicities and may lead to complications such as growth retardation, cardiomyopathy, and endocrine dysfunction (216–218). Although many promising results have been reported, these significant side effects restrict the use of iron chelators in clinical practice. Recently, Xu et al. synthesized PEGylated DFO using polyethylene glycol (PEG) as a delivery carrier. Metabolic studies showed that the stability of PEGylated DFO is markedly enhanced, and its half-life is 20 times longer than that of DFO. *In vitro* experiments further revealed that PEGylated DFO effectively reduced iron deposition and neuronal degeneration while promoting neurological recovery in stroke model rats (219). Similar therapeutic effects can be achieved at lower DFO doses, suggesting a potential strategy for iron overload disorders. Despite the favorable outcomes observed with PEGylated DFO derivatives in animal models, these formulations have not been tested in clinical trials for IS. Accordingly, the clinical benefit of DFO in patients with IS remains unclear, and further translational research is warranted.

### 5.3 YC-1

3-(5’-Hydroxymethyl-2′-furyl)-1-benzyl indazole (YC-1) is a reversible activator of soluble guanylate cyclase. It effectively suppresses HIF-1α activity by enhancing the hydroxylation of its proline residues, thereby interfering with hypoxic pathological processes. YC-1 also reduces the transcriptional activity of HIF-1α by disrupting its dimerization with HIF-1β. Notably, in a rat model of tMCAO, intraperitoneal injection of YC-1 2 h before ischemia followed by sacrifice at 24 h after reperfusion significantly reduced HIF-1α and NLRP3 protein expression, decreased cerebral infarct volume, and attenuated neurological deficits (220). In contrast, Zhang et al. administered YC-1 intravenously twice (at 24 h and 30 min before ischemia) in a rat tMCAO model (90 min of ischemia followed by 24 h of reperfusion). They found that YC-1 significantly suppressed HIF-1α, EPO, and GLUT-3 protein expression, while leading to a marked increase in infarct volume (140). Furthermore, Sun et al. used a rat tMCAO model (2 h of ischemia) with two intravenous injections of YC-1 given at 24 h and 30 min before ischemia, and harvested tissue at 10 min after reperfusion. Their results showed that YC-1 significantly reduced BBB leakage and preserved BBB integrity (157).

Opposite to the above findings, in a rat tMCAO model, continuous intravenous infusion of YC-1 for 7 days significantly inhibited HIF-1α, VEGFA, and EPO protein expression, while increasing infarct volume and exacerbating neurological deficits (139). Moreover, Qin et al. administered YC-1 intraperitoneally for 14 consecutive days in a rat tMCAO model and observed a significant increase in cerebral infarct volume and neuronal death, accompanied by aggravated astrocyte activation (221). These findings suggest that YC-1 administration may be beneficial during the early phase of IS (< 24 h) but becomes detrimental beyond 24 h. Therefore, the timing of YC-1 administration must be carefully selected to avoid interfering with neural repair processes during the late stage of IS.

### 5.4 Propofol

Propofol is the predominant intravenous general anesthetic used in emergency surgeries (222), primarily acting through activation of the inhibitory neurotransmitter γ-aminobutyric acid (223). One study showed that propofol reduced cerebral infarct volume and promoted neurological functional recovery in mice with IS (224). Furthermore, in OGD/R models, propofol downregulated YTHDF1 and BECN1 expression by reducing HIF-1α levels, ultimately inhibiting ferroptosis (191). However, current research on propofol’s role in regulating ferroptosis after IS remains limited. Propofol is not a specific HIF-1α modulator, and its neuroprotective effects may involve multiple pathways. Additionally, propofol is associated with adverse effects in clinical practice, including dose-dependent hypotension and allergic reactions, and prolonged administration may result in metabolic disorders (225). These factors may hinder its clinical translation as a therapeutic strategy for IS.

### 5.5 Natural products

Natural products typically possess multiple pharmacological activities, including antioxidant, anti-inflammatory, and anti-ferroptotic effects. Their regulation of HIF-1α is mostly indirect, making them potential neuroprotective agents (226, 227). However, research has largely focused on rodent models, and clinical studies in patients with IS remain limited. Although these compounds are generally regarded as safe, adverse effects may still occur (228). Therefore, their safety and potential side effects require further evaluation during clinical translation.

#### 5.5.1 Wedelolactone

Wedelolactone (Wel) is a small-molecule furanocoumarin derived from the medicinal plant *Eclipta prostrata* L., with pharmacological activities including hepatoprotective, anti-inflammatory, hypoglycemic, and neuroprotective effects. Wel has been studied in various diseases, including coronary heart disease, liver disorders, and neurological conditions (229). Previous studies demonstrated the neuroprotective properties of *Eclipta prostrata* L. extracts on rat brain neurons (230, 231). Recently, Tao et al. showed that Wel treatment could activate HIF-1α and promote recovery of damaged cells. Activated HIF-1α inhibited neuronal ferroptosis by directly stimulating SLC7A11 and GPX4, thereby improving brain injury in tMCAO model mice (2 h ischemia and 7 days reperfusion) (117).

#### 5.5.2 Chrysin

Chrysin is a natural flavonoid found in propolis, Scutellaria baicalensis Georgi, and other botanical sources. Due to its antioxidant and neuroprotective properties, chrysin has been investigated in multiple neurological conditions, including IS, Alzheimer’s disease, and Parkinson’s disease (232, 233). Previous studies indicated that chrysin promotes cell proliferation and suppresses inflammatory responses in PC12 cells subjected to OGD/R. Recently, Shang et al. demonstrated that in a rat tMCAO model (2 h ischemia and 22 h reperfusion), chrysin inhibited HIF-1α nuclear translocation, which impeded CP expression and downregulated its downstream targets Tfr1 and ACSL4, thereby alleviating ferroptosis (234).

#### 5.5.3 Protocatechuic acid

Protocatechuic acid (PCA) is a phenolic acid commonly found in foods such as olive, rice, onion, and grape, as well as in herbs including *Salvia miltiorrhiza* and Alpinia oxyphylla (235–238). PCA exhibits anti-inflammatory, antioxidant, antimicrobial, antiviral, analgesic, and anti-atherosclerotic activities (238, 239). Additionally, PCA has significant neuroprotective properties and is considered a potential therapeutic agent for neurological diseases, including Alzheimer’s disease and Parkinson’s disease. A recent study reported that PCA protects against hypoxic–ischemic encephalopathy by activating the HIF-1α/VEGF pathway through VEGF nuclear translocation in cerebrovascular endothelial cells, improving neurological scores and reducing infarct volume, thereby suppressing ferroptosis via ACSL4 downregulation (202). However, this study was conducted in a hypoxic–ischemic encephalopathy model rather than IS, providing only indirect evidence for IS therapy. Further investigations in IS models are warranted.

#### 5.5.4 Hydroxysafflor yellow A

Hydroxysafflor yellow A (HSYA) is the primary active compound of safflower, and possesses antithrombotic and neuroprotective properties (240). Previous studies showed that HSYA alleviated brain damage in tMCAO rats (2 h ischemia and 72 h reperfusion) by upregulating HIF-1α expression (241). Wu et al. also reported that HSYA improved brain damage in tMCAO rats by significantly increasing FPN1 and GPX4 levels, thereby preventing neuronal ferroptosis (16). HSYA further inhibits NCOA4-mediated ferritinophagy, reducing ferritin degradation and free iron release, which restores GPX4 activity, attenuates LPO, and ultimately suppresses neuronal ferroptosis in tMCAO rats (16). Additionally, HSYA alleviates cardiac ischemia and suppresses ferroptosis via activation of the HIF-1α/SLC7A11/GPX4 signaling pathway in a myocardial ischemia–reperfusion injury model (242). Collectively, these findings provide indirect evidence, and we speculate that HSYA may inhibit ferroptosis by modulating HIF-1α, representing a promising avenue for future mechanistic studies.

Regarding safety, a phase I clinical trial demonstrated that HSYA was generally well tolerated in healthy subjects. All adverse events were mild, with common side effects including increased heart rate, reduced platelet aggregation, and mild elevations in serum total bilirubin and transaminase levels (228). Therefore, further monitoring of the adverse events is warranted in future studies when HSYA is applied to IS.

## 6 Conclusion

This review summarizes the dual role of HIF-1α in ferroptosis after IS and its potential therapeutic implications. HIF-1α can upregulate proteins such as FPN1, SLC7A11, and GPX4 to promote iron export and enhance antioxidant capacity, thereby suppressing ferroptosis and alleviating neuronal damage. Conversely, it can also facilitate ferroptosis by inducing Tfr1 expression, increasing free iron accumulation, or aggravating LPO via the HIF-1α/YTHDF1/BECN1 pathway. These divergent outcomes may be closely related to dynamic changes in HIF-1α expression resulting from variations in ischemia duration and injury severity.

Notably, limited clinical studies indicate that higher HIF-1α levels are associated with more severe neurological damage (113, 172). Moreover, patients with IS exhibit abnormalities in peripheral blood hepcidin (59), as well as in serum GPX4 and SLC7A11 levels (86), which correlate closely with disease severity, suggesting their potential as prognostic markers. However, clinical investigations examining the relationship between dynamic HIF-1α changes and ferroptosis-related indicators remain scarce. Future research should aim to define the “threshold” at which HIF-1α shifts from inhibiting to promoting ferroptosis, considering factors such as injury extent, ischemia duration, specific serum concentration ranges, and the expression ratio of pro-apoptotic to pro-survival target genes. Although HIF-1α has shown considerable promise as a regulator of ferroptosis in IS, some therapeutic strategies still encounter challenges in clinical translation, and their adverse effects warrant ongoing monitoring.
